# Phytochemistry and Biological Profile of the Chinese Endemic Herb Genus *Notopterygium*

**DOI:** 10.3390/molecules29143252

**Published:** 2024-07-09

**Authors:** Zhikang Tang, Renlin Zheng, Ping Chen, Liangchun Li

**Affiliations:** School of Life Science and Engineering, Southwest University of Science and Technology, Mianyang 621010, China; tzkyaan@swust.edu.cn (Z.T.); zhengrenlin@swust.edu.cn (R.Z.); pingchen@swust.edu.cn (P.C.)

**Keywords:** *Notopterygium incisum*, *Notopterygium franchetii*, phytochemistry, biological activity, pharmacology, Qianghuo

## Abstract

*Notopterygium*, a plant genus belonging to the Apiaceae family, is utilized in traditional Chinese medicine for its medicinal properties. Specifically, the roots and rhizomes of these plants are employed in phytotherapy to alleviate inflammatory conditions and headaches. This review provides a concise overview of the existing information regarding the botanical description, phytochemistry, pharmacology, and molecular mechanisms of the two *Notopterygium* species: *Notopterygium incisum* and *N. franchetii*. More than 500 distinct compounds have been derived from these plants, with the root being the primary source. These components include volatile oils, coumarins, enynes, sesquiterpenes, organic acids and esters, flavonoids, and various other compounds. Research suggests that *Notopterygium incisum* and *N. franchetii* exhibit a diverse array of pharmacological effects, encompassing antipyretic, analgesic, anti-inflammatory, antiarrhythmic, anticoagulant, antibacterial, antioxidant, and anticancer properties on various organs such as the brain, heart, digestive system, and respiratory system. Building activity screening models based on the pharmacological effects of *Notopterygium* species, as well as discovering and studying the pharmacological mechanisms of novel active ingredients, will constitute the primary development focus of *Notopterygium* medicinal research in the future.

## 1. Introduction

*Notopterygium*, a member of the Apiaceae family, is a significant plant genus in traditional Chinese medicine. Within the Chinese pharmacopeia, the rhizomes of two specific species belonging to the *Notopterygium* genus are commonly referred to as “Qianghuo” [1]. These species are *Notopterygium incisum Ting ex H. T. Chang* (NI) and *Notopterygium franchetii H. de Boiss* (NF). The first known record of it can be found in *Shennong Ben Cao Jing*, a significant text on ancient Chinese herbal medicine. In traditional Chinese medicine, this substance is known for its bitter, aromatic, and slightly spicy taste. It possesses unique qualities that make it useful for therapeutic purposes. The rhizomes and roots of this herb have historically been utilized to treat inflammatory diseases like rheumatoid arthritis, as well as to alleviate the pain associated with headaches [2]. Additionally, they have been used as a diaphoretic and to treat colds [3].

The main chemical components isolated in traditional Chinese medicine Qianghuo include volatile oils, terpenes, coumarins, sugars and glycosides, phenolic acids, polyene alkynes, alkaloids, etc. [4]. Modern pharmacological research has shown that these chemical components have anti-inflammatory, antibacterial, antioxidant, antiviral, anti-cancer cell proliferation, antipyretic and analgesic activities, and have significant effects on the cardiovascular, digestive, respiratory, and central nervous systems [5].

Chang et al. reviewed the ethnopharmacology, phytochemistry and pharmacology of NI in 2017 [3]. Since then research has expanded on the knowledge of the phytochemical and pharmacological areas of NI and NF. This review seeks to provide a concise summary of recent phytochemical and biological studies on the two *Notopterygium* species, focusing on examples published since the publication of the last major review in 2017. This work deals with studies of botanical descriptions, phytochemical composition, pharmacological activity, molecular mechanisms, and traditional applications of various plant parts of NI and NF. The primary focus is on the roots and rhizomes, which are commonly used in traditional medicine. However, studies on the seeds, although less popular, are also included.

## 2. Botanical Description

### 2.1. Botanical Systematics

*Notopterygium* H. Boissieu (Apiaceae) is unique to China and comprises six species, as documented in *Flora of China* [6,7]. Among these, three have a relatively broad regional distribution (*Notopterygium incisum* C. C. Ting ex H. T. Chang, *Notopterygium franchetii* H. de Boissieu, and *Notopterygium oviforme* R. H. Shan), while three have a narrow endemic distribution (*Notopterygium forrestii* H. Wolff, *Notopterygium pinnatiinvolucellatum* F. T. Pu, and *Notopterygium tenuifolium* M. L. Sheh and F. T. Pu) [8]. Based on molecular evidence presented by Yang et al., four lineages of species belonging to the genus *Notopterygium*: *Notopterygium forrestii*, *Notopterygium franchetii*, *Notopterygium incisum*, and *Notopterygium oviforme* [9] are identified. 

Jia et al. employed nuclear expressed sequence tag–simple sequence repeat and large-scale single nucleotide polymorphism variation data, along with niche analysis, to examine the potential separate instances of hybridization in *Notopterygium* [6]. Population genomic research revealed that the four species under study are genetically distinct and that *Notopterygium forrestii* and *Notopterygium oviforme* emerged through hybridization. Niche studies revealed that the differentiation in ecological niches might have facilitated and sustained the reproductive isolation between hybrid species [10]. Liu et al. confirmed that the environmental ecological niches of the four species are comparatively distinct [6]. *Notopterygium forrestii* inhabits elevated grasslands and forest margins ranging from 4000 to 4300 m in the southeastern Qinghai–Tibetan Plateau (QTP) in China [8]. Conversely, *Notopterygium oviforme* is found in the Qingling mountain regions of central and western China at considerably lower altitudes (1700 to 3000 m). The two remaining species, *Notopterygium incisum* and *Notopterygium franchetii*, are parapatrically distributed along the eastern QTP, albeit at distinct elevations (3000–4700 m for *Notopterygium incisum* and 2000–3300 m for *Notopterygium franchetii*, respectively) [6].

### 2.2. Botanical Characteristics

**Figure 1:**
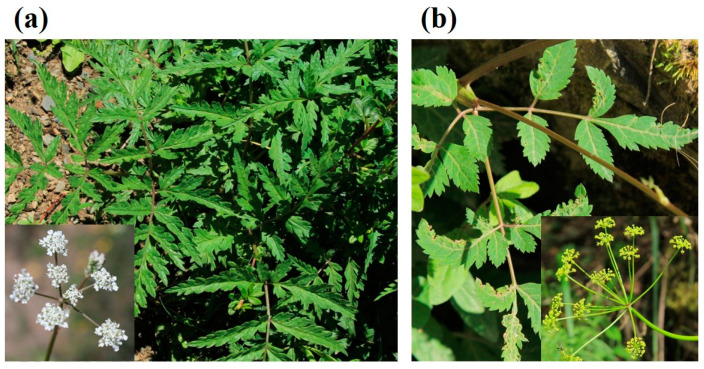
The *Notopterygium* plant and its flowers: (**a**) NI; (**b**) NF (picture taken by authors).

The *Notopterygium* plant is distinguished by its many flowers that include calyx teeth with an ovate-triangular shape [11] (Figure 1). The petals, which have a maximum count of five, exhibit a white hue. Their shape is obovate and their apex is obtuse and concave. The blooming season lasts from July to September, while fruit development takes place from August to October. NI or NF is a perennial plant that grows between 60 and 150 cm in height. The sturdy rhizome exhibits either a cylindrical or irregularly shaped protuberance. The underground section of NI or NF can be divided into three distinct components. The first is the rhizome, which is distinguished by its compact internodes. The nodes demonstrate a significant density and possess a configuration that closely resembles that of silkworms, with measurements ranging from 4 to 13 cm in length and 0.6 to 2.5 cm in diameter. The second component is made up of enlarged internodes 15–40 cm in length and 0.5–1.5 cm in diameter. The third component is the roots, which are located at the terminus of the plant and sometimes at rhizome nodes. The roots range from 6 to 14 cm in length, and have a diameter of 0.5 to 1.5 cm. The underground section displays a brown color and releases a unique strong scent. The stems have a tubular and cylindrical shape, and are a clear lavender color with straight vertical lines. The basal leaves have extended petioles that extend into a membranous sheath, from the base to the sides. In addition, the leaf blade has a ternate-3-pinnate arrangement, comprising of three to four pairs of leaflets.

## 3. Phytochemical Composition

Currently about 500 different substances have been extracted from *Notopterygium* species, with the majority originating from the roots of either NI or NF. The substances can be categorized into distinct groups including volatile oils, coumarins, enynes, sesquiterpenes, organic acids and esters, flavonoids, and other substances.

### 3.1. Volatile Oils 

Volatile oil or essential oil (EO) is an important active ingredient in *Notopterygium* [12,13], and the is limited content about EOs in Qianghuo in the Chinese Pharmacopoeia [1]. Evidently, there were significant differences in the components of EOs obtained from different places and periods of growth. Wedge et al. found that major components of NI root oils were limonene, α-pinene, and β-pinene [14]. Bi et al. found that three main compounds of NI root oils were β-pinene, α-pinene and γ-terpinene [13]. Wang et al. found that limonene (18.42%) and γ-terpinene (15.03%) were the major components of EOs from the aerial parts of NI, followed by β-phellandrene (9.47%), and carotol (7.99%) [13]. 

Although supercritical fluids extraction (SFE) methods have been reported to improve the extraction rate of volatile oils, there are significant differences between the composition of the volatile oil components obtained in this way and those obtained by water distillation extraction (WDE) methods [15,16]. Three components with the highest content in volatile oil by WDE are β-pinene, α-pinene and terpene—their contents reaching 35.608%, 30.424%, and 10.304%, respectively [17]. When extracting by SFE, 6-allyl-4-methoxy-1,3-benzodioxole, β-pinene, 4-vinyl-2-methoxy-phenol, α-pinene, borneol acetate, and terpenes are the main components, with contents of 21.572%, 16.412%, 8.382%, 7.290%, 6.588%, and 6.228%, respectively [18].

### 3.2. Coumarins

**Figure 2:**
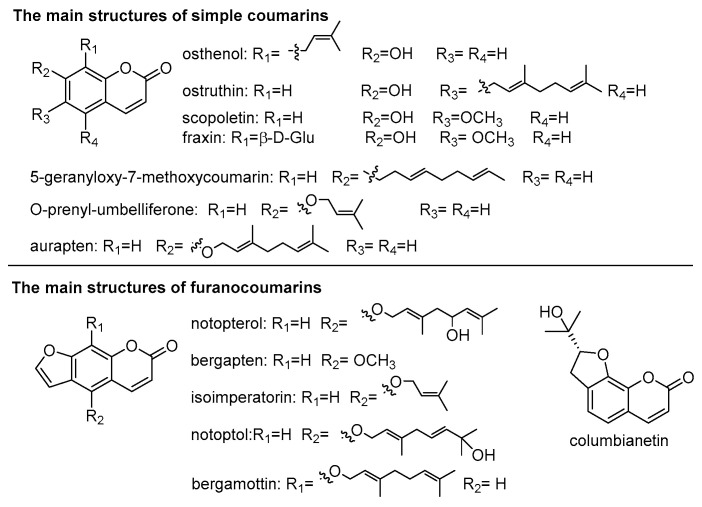
The main structures of coumarins in NI and NF.

**Figure 3:**
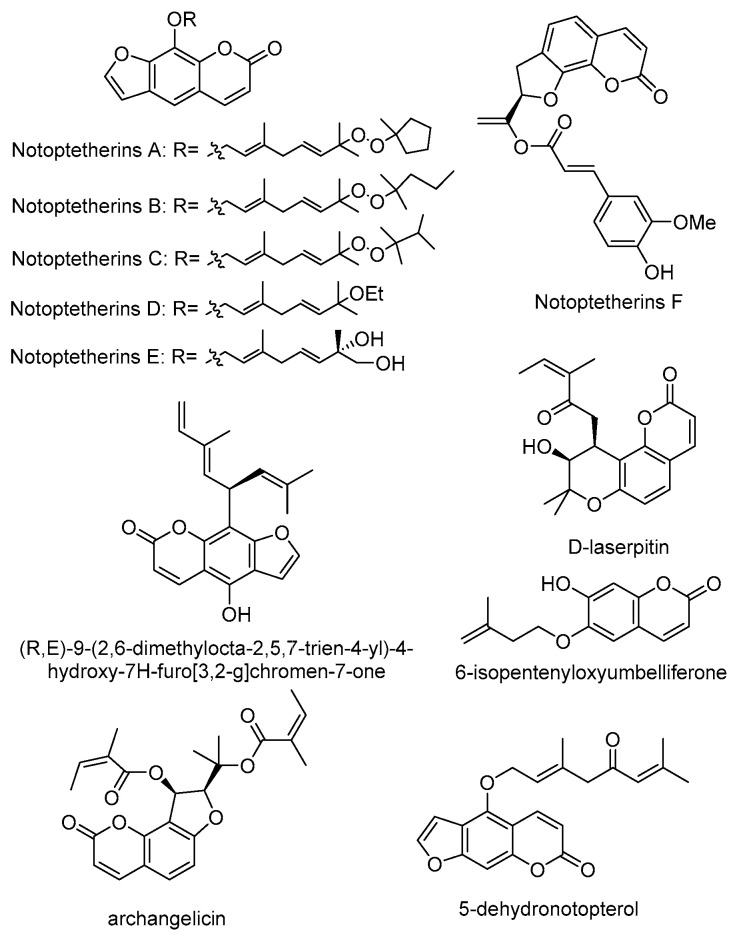
The structures of newly discovered coumarins and known coumarins recently isolated from NI and NF for the first time.

Coumarins are chemical compounds made of fused benzene and α-pyrone rings found in many plants [19]. Coumarins have a diverse range of biological characteristics, including antibacterial [20], antiviral [21,22], anti-inflammatory [23], anticancer [24], antidiabetic [25], antioxidant [26], and enzyme inhibitory effects [27,28]. The roots, rhizomes, and seeds of NI and NF contain coumarins, which are the earliest and most commonly discovered chemical components of plants. The coumarins in *Notopterygium* species can be divided into two types: simple coumarins and furanocoumarines (Figure 2). The simple coumarins are mainly derivatives of −OH substituents or −OCH_3_ substituents in benzyl of coumarins and alkenyl groups connected by oxygen bridges, such as osthenol [29,30], ostruthin [29,31], scopoletin [31,32], 5-geranyloxy-7-methoxycoumarin [31,32], fraxin [33], O-prenyl-umbelliferone [34] and aurapten [35]. The key structures of furanocoumarins are various from angular to psoralen and nodakenetin [36]. Among them, nodakenin [33,37,38], notopterol [37], bergapten [37,39,40], isoimperatorin [39,40,41], notoptol [37], bergamottin [39,40,41], and columbianetin [42] are the representative compounds in NI and NF (Figure 2). According to reports, the content of notopterol is higher in NI, while it is lower in NF [43]. Conversely, it is reported that the content of nodakenin is higher in NF, while lower in NI [2,44]. In recent years, continuing phytochemical research has been conducted on *Notopterygium* species. Several new coumarins were found in NI, such as Notoptetherins A-F [35], 5-dehydronotopterol [45] and (R,E)-9-(2,6-dimethylocta-2,5,7-trien-4-yl)-4-hydroxy-7H-furo[3,2-g]chromen-7-one [46] (Figure 3). Besides these, D-laserpitin [47], archangelicin [47] and 6-isopentenyloxyumbelliferone [45] were isolated from the roots and rhizomes of NI for the first time. Umbelliferone, oxypeucedanin, bergapten, O-prenyl-umbelliferone and phellopterin were detected in the seeds of NF; in contrast these compounds were detected at trace levels in NI [34,48]. Moreover, the contents of kynurenic acid and oxypeucedanin hydrate were higher in the seeds of NI than in those of NF.

### 3.3. Polyene-Alkyne

**Figure 4:**
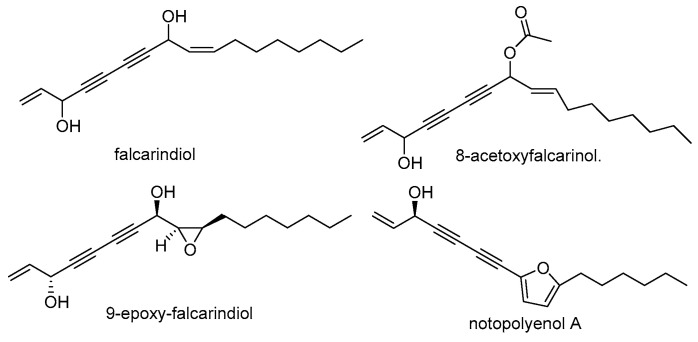
The representative structures of polyene-alkyne from NI and NF.

Alkyne-containing natural products are significant compounds that have a broad distribution in microorganisms and plants [49]. A significant number of them exhibit encouraging biological properties, including anti-tumor, anti-parasitic, anti-malarial, and anti-HIV actions [50]. The polyene alkyne compounds found in NI and NF generally have two alkyne bonds in each structure, mainly derived from the roots and rhizomes. Most of them are derivatives of falcarindiol such as 9-epoxy-falcarindiol [51] and 8-acetoxyfalcarinol (Figure 4). Liu et al. identified eleven new polyene alkyne compounds named notoethers A–H and notoincisols A–C from the roots and rhizomes of NI [52]. The newly discovered compounds possess distinct chemical diversity due to their characteristic combination of falcarindiol with sesquiterpenoid or phenylpropanoid moieties. Phytochemical investigation by the Tu group on the roots and rhizomes of NI has led to the recent isolation of a new polyacetylene—notopolyenol A—along with thirteen known analogues [53]. The researchers utilized synthetic techniques to produce enantiomers and effectively to differentiate and identify the absolute configuration of a novel molecule using HPLC chiral separation and ECD spectroscopic analysis.

### 3.4. Phenolics and Flavonoids

**Figure 5:**
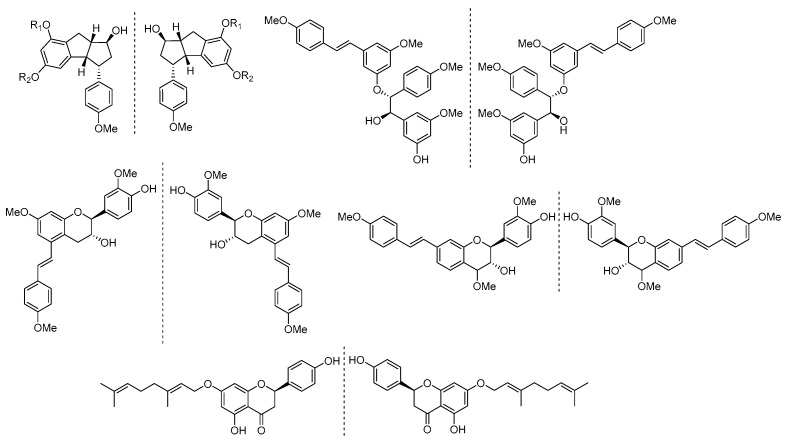
The structures of enantiomers isolated from the roots and rhizomes of NI.

Flavonoids and other phenolic compounds are commonly known as plant secondary metabolites that have an aromatic ring bearing at least one hydroxyl group [54,55,56]. Nine new stilbene derivatives were isolated from the roots and rhizomes of NI in 2022. Among these eight compounds were four pairs of enantiomers, two of the enantiomers featuring an unprecedented 1-benzyl-2-methyl-indane skeleton in natural source (Figure 5) [57]. The absolute configurations of new compounds were determined by quantum chemical calculations of the electronic circular dichroism (ECD) spectra, comparison of the experimental ECD data with those reported, and by chemical methods. Eight known flavonoids were obtained from the roots and rhizomes of NI and NF, and contain two pairs of enantiomers.

### 3.5. Others

Alkaloid [34,58], terpenoid [32,33], glycosides [33] and sterols [59] were also isolated from *Notopterygium* species, mostly from the seeds of NI. Detailed information about structures can be found in the review by Chang [3], after which no new relative structures have been reported.

## 4. Biological Activity

*Notopterygium* plants have a wide range of pharmacological properties, including antipyretic, analgesic, anti-inflammatory, antiarrhythmic, anticoagulant, antibacterial, antioxidant, and anticancer effects on the brain, heart, digestive system, and respiratory system (Table 1). The complex pharmacological properties of NI or NF do not take effect independently. These antibacterial, antipyretic and analgesic properties not only regulate the immune system, but also directly or indirectly have symptomatic and curative anti-inflammatory effects [60,61]. Relief from gastric ulcer, diarrhea and asthma are all related to the regulation of inflammatory factors [62]. Regulating the immune system can produce anti-cancer effects [63]. The analgesic effect of NI targets pain caused by inflammation and ischemia [64]. Antioxidants and anticoagulants can alleviate pain caused by cerebral and myocardial ischemia [65]. Since the Ming dynasty, Qianghuo has been an integral component in a considerable number of Chinese prescriptions for the treatment of ailments [66,67].

### 4.1. Anti-Inflammatory

Several systemic inflammatory pathways, including interleukin (IL), nuclear factor-kappa B (NF-κB), tumor necrosis factor-alpha (TNF-α), and P38 mitogen-activated protein kinase-dependent pathways rely on oxidative stress [91]. The interplay of these molecules within an intricate network regulates the inflammatory processes of the body [92]. Notopterol serves as a quality control indicator for NI, exhibiting anti-inflammatory, antioxidant, and analgesic properties. In addition, notopterol has been utilized for an extended period to address joint ailments [68]. A study using human C28/I2 cells suggested that notopterol down-regulated the hypersecretion of inflammatory mediators and alleviated the degradation of the extracellular matrix (ECM) [68]. In addition, notopterol decreased the overproduction of reactive oxygen species (ROS) [93] and chondrocyte apoptosis through the nuclear factor erythroid-2-related factor 2 (Nrf2)-signaling pathway. Zhou et al. showed that notopterol could inhibit osteoclastogenesis, thereby limiting alveolar bone loss in vivo. In vitro results demonstrated that notopterol administration inhibited synthesis of inflammatory mediators such as IL-1β, IL-32, and IL-8 in LPS-stimulated human gingival fibroblasts [69]. Mechanistically, notopterol inhibits activation of the NF-κB signaling pathway, which is considered a prototypical proinflammatory signaling pathway. Collectively these results raise the possibility that notopterol relieves periodontal inflammation by suppressing and activating the NF-κB and PI3K/AKT/Nrf2-signaling pathways in periodontal tissue, suggesting its potential as an efficacious treatment therapy for periodontitis.

Huang et al. indicated that notopterol enhanced the survival rate and improved the function of the right ventricle, while also decreasing the systolic pressure in the right ventricle of rats with pulmonary arterial hypertension produced by monocrotaline [70]. In addition, notopterol effectively decreased the enlargement of the right ventricle and the formation of scar tissue. It also alleviated the restructuring of blood vessels in the lungs and the thickening of muscles caused by MCT. Furthermore, notopterol reduced the activity of pro-inflammatory factors IL-1β and IL-6, as well as PCNA, in the lungs of rats with pulmonary arterial hypertension (PAH). Additional research emphasized the potential of notopterol as a therapeutic agent in the treatment of osteoarthritis (OA), specifically in protecting cartilage from damage caused by inflammation, by inhibiting pyroptosis [71]. The research showed that notopterol effectively decreased the levels of IL-18 and TNF-α in inflamed cells. It also reduced the formation of ROS after inflammation and blocked the JAK2/STAT3 signaling pathway. As a result, chondrocytes were protected from inflammation.

The main pathological process responsible for obstructive sleep apnea syndrome (OSAS) is chronic intermittent hypoxia (CIH), which is closely associated with systemic inflammation [72]. Pterostilbene and notopterol have demonstrated potential therapeutic effects on OSAS. The PTGS2 and estrogen receptor alpha (ESR1) genes were linked to OSAS. A pathway enrichment study specifically targeted the NF-κB, apoptosis, and HIF-1A pathways. Pterostilbene and notopterol reduced the levels of IL-6, TNF-α, and PGE2 in response to CIH. An upregulation of PTGS2 levels led to the activation of the NF-κB pathway. Pterostilbene facilitated the process of proteasome-mediated ubiquitination of PTGS2 protein, resulting in a decrease in PTGS2 levels and subsequent blocking of the NF-κB pathway.

Besides notopterol, other structures also showed anti-inflammatory bioactivity. For example, Wu et al. found that 4-methyl-3-trans-hexenylferulate, (-)-boroylferulate, 4-methoxyphenyl ferulate, and phenylyl ferulate in NI can significantly inhibit NO production with IC50 values of 1.01, 4.63, 2.47, and 2.73 μM, respectively, which were more effective than positive control (L-NIL) with IC50 values of 9.37 μM [73]. Using activity-guided isolation, Blunder et al. tested the inhibitory activity of certain compounds on nitric oxide production in RAW 264.7 mouse macrophages using the Griess assay [74]. They found that 3-hydroxy allyl polyacetylenes exhibited significant activity. The Tu group also evaluated nitric oxide inhibitory effects on RAW264.7 cells induced by LPS for the isolated furocoumarins [35] and phenolic constituents [57]. They found that two furocoumarins and five flavonoids notoflavinols A and B, (2R)-5,4′-dihydroxy-7-O-[(E)-3,7-dimethyl-2,6-octadienyl]flavanone, and their hydrogenated products showed moderate inhibitory activities with IC50 values in the range of 6.2–20.6 μM.

Li et al. used three compounds including nodakenetin, isoimperatorin, and pregnenolone from NF to treat chronic inflammation pain in C57BL/6 mice [75]. It was discovered that Nodakenetin effectively reduced CFA-induced inflammatory pain, but did not have a substantial therapeutic impact. However, isoimperatorin and pregnenolone did not alleviate the inflammatory discomfort generated by CFA. In terms of its mechanism, nodakenetin inhibited the activation of the NF-κB pathway and the phosphorylation of IκB*α* in HEK293T cells, which was stimulated by IL-1β. In addition, the application of Nodakenetin reduced the levels of IL-6, TNF-α, and IL-1β expression in macrophages obtained from mouse bone marrow.

Ohnuma et al. found that falcarindiol isolated from NI extract activated the Nrf2/ARE pathway and induced cytoprotective enzymes, which might be useful as chemopreventive agents [76]. Atanasov et al. investigated extracts of the underground parts of NI and observed significant PPARγ activation using a PPARγ-driven luciferase reporter model [51]. Activity-guided fractionation of the dichloromethane extract led to the isolation of six polyacetylenes, which displayed properties of selective partial PPARγ agonists in the luciferase reporter model. They found that falcarindiol bound to the purified human PPARγ receptor with a Ki of 3.07 µM. P2Xs are useful targets for inflammatory pain therapy.

The volatile oils and extracts of NI and NF showed anti-arthritic capacity in the traditional treatment of rheumatoid arthritis (RA) [13]. Recent research found that the extraction of polysaccharides from NF exhibited strong antioxidant activity in vitro and potent anti-inflammatory activity in zebrafish embryos [94]. Wang et al. found that the extract of NI significantly down-regulates P2X1, P2X3, P2X4, P2X5, and P2X7 to inhibit FCA-induced RA in rats [77]. Among these, P2X3 showed the most significant down-regulation. Another study by Liu demonstrated that ethyl acetate extracted from NI can regulate NLRP3, pro-Caspase-1, Caspase-1, and CD11b in the ankle joints of AA rats [78]. NI can effectively mitigate the inflammatory response generated by lipopolysaccharide (LPS) in RAW264.7 cells by alleviating mitochondrial damage, decreasing mitochondrial DNA levels and mitochondrial ROS, and suppressing the activation of the NLRP3 inflammasome. Research has demonstrated that NI root extract (NRE) can ameliorate epilepsy and improve cognitive function in mice with Alzheimer’s disease (AD). Its potential action may involve the reduction of inflammation and enhancement of antioxidant defense [79].

NI has been used as an ingredient in more than thirteen Chinese prescriptions in the treatment of diseases [3]. Some formulae containing NI have been shown to have anti-inflammatory and analgesic effects. For example, *Clematis chinensis* Osbeck/NI (CN) is an efficacious traditional Chinese herb. Pan found out that the herb couple CN significantly down-regulated levels of TNF-α, IL-6, and VEGF and shows evident anti-arthritic effects in AIA rats [80]. San Hua Tang (SHT) is a representative formula for treating stroke, which is composed of *Rheum palmatum* L., *Magnolia officinalis* Rehder & E.H. Wilson, *Citrus assamensis* S. Dutta & S.C. Bhattacharya, and NI. The results in ischemic stroke (IS) treatment by SHT show that it regulates gut microbiota, inhibits pro-inflammatory factors in rats with IS, alleviates inflammatory injury of the blood-brain barrier, and plays a protective role in the brain [81]. Researchers believe that the anti-inflammatory and anti-apoptotic effects of NI demonstrate efficacy in the formula [95].

### 4.2. Anti-Tumor

Some compounds obtained from *Notopterygium* have varying degrees of inhibitory effects on the proliferation of various cancer cells. For example, Wu et al. reported that notopol, notopterol, 5-[(2E,5Z)-7-hydroxy-3,7-dimethyl-2,5-octadienoxy]psoralene, and 5-[(2,5)-epoxy-3-hydroxy-3,7-dimethyl-6-octenoxy]psoralene showed significant antiproliferative activity against HepG-2 and C6 cancer cell lines, with IC_50_ values of 7.7–24.8 mg/mL [82]. Structure–activity relationship (SAR) research showed that the presence of a free hydroxy at the lipophilic side chain linked to C-5 of the linear furocoumarins was essential for their in vitro antiproliferative activity.

Zhou et al. found out that notopterol effectively decreased the concentrations of cytokines (iNOS, TNF-α, IL-6, and IL-β) and suppressed the activity of the STAT3/NF-κB signaling pathway in brain tissues surrounding the tumor and in a microglial cell line (BV2 cells) treated with GL261 conditioned media (GCM) [86]. The results showed that notopterol had anti-glioma effects which decreased STAT3 activity and alleviated neuropsychiatric symptoms by inhibiting tumor-associated inflammation through modulation of the STAT3/NF-κB pathway in mice with glioma.

Huang et al. demonstrated that notopterol significantly suppressed the viability, migration, and invasion capacity of the human HCC HepJ5 and Mahlavu cell lines by disrupting ATF4 expression, inhibiting JAK2 activation, and down-regulating the GPX1 and SOD1 expression in vitro [83]. Notopterol also dose-dependently suppressed the expression of vimentin (VIM), snail, b-catenin, and N-cadherin in HCC cells. In another study, Wang et al. found that notopterol directly bound Janus kinase (JAK)2 and JAK3 kinase domains to inhibit JAK/signal transducers and activators of transcription (JAK-STAT) activation, leading to reduced production of inflammatory cytokines and chemokines [84].

The inhibition of the oncogenic pathway induced by IL-17 represents an innovative approach in the management of lung cancer [96]. Inthanon demonstrated that treatment with notopterol substantially decreased the phosphorylation of Akt, JNK, ERK1/2, and STAT3 that was activated by IL-17 [85]. This, in turn, resulted in a diminished level of transcriptional activity for NF-κB and AP-1. The findings indicate that notopterol inhibits the proliferation and invasion of A549 cells induced by IL-17 by modulating EMT and inhibiting the MAPK, Akt, STAT3, AP-1, and NF-κB signaling pathways.

Li et al. evaluated the in-vitro cytotoxicity of extracts from NI and found that strustin had a significant activity on PANC-1 (PC_50_ = 7.2 μmol/L) and PSN-1 (PC_50_ = 7.8 μmol/L) [87]. Additionally, two known compounds ostruthin and (-)-bornyl ferulate obtained from NI or NF both induced comparable neurite-like structures in twenty percent of rat PC12 cells at 2 micrograms/mL, and showed cytotoxicity at concentrations higher than 3 mg/mL.

These research findings suggest that the chemicals derived from *Notopterygium* possess significant promise for application in cancer treatment. Specifically, they demonstrate efficacy in suppressing the growth of cancer cells, modulating signaling pathways, and mitigating inflammation associated with tumors. Further investigation is warranted to explore the underlying processes by which these chemicals exert their effects, as well as their suitability and safety for use in clinical therapy.

### 4.3. Antibacterial and Antifungal

Wang et al. investigated the composition, antibacterial activity, and cytotoxicity of essential oil (EO) from aerial parts of NI [13]. The antibacterial activity and mechanism study showed that the diameters of inhibition zone (DIZs) of NI-EO against *E. coli* and *S. aureus* were 14.63 and 11.25 mm and that the minimum inhibitory concentrations were 3.75 and 7.5 μL/mL, respectively. NI-EO not only caused intracellular biomacromolecule leakage and cell deformation by destroying bacterial cell wall integrity and cell membrane permeability, but also degraded the mature biofilm. The low toxicity of NI-EO was demonstrated in an assay on bovine mammary epithelial cells. These results suggest that NI-EO was mainly composed of monoterpenes and sesquiterpenes, had excellent antibacterial activity and showed low levels of cytotoxicity [97]. It is expected to be applied as a natural antibacterial agent in the future.

An elucidation was performed on nine antifungal secondary metabolites from an NI ethyl acetate extract: five linear furocoumarins, two phenylethyl esters, one falcarindiol, and one sesquiterpenoid [46]. The report described the antifungal properties of the purified compounds against Apple Fruit Pathogens (Colletotrichum *gloeosporioides* and Botryosphaeria dothidea), with MIC values varying between 8 and 250 mg L^−1^.

### 4.4. Other Research

Research conducted over 20 years ago illustrated the anti-viral and anti-arrhythmic effects of NI extracts. Zhao and Gao demonstrated that an extract of NI had a potential anti-AD effect via the inhibition of the Aβ cascade, tau pathology and neuroinflammation in vitro and in vivo [88]. Additionally an extract of NI showed potential therapeutic effects on ovariectomy-induced osteoporotic and thrombus rats. However, these studies have not identified specific molecular structures and it is difficult to conduct in-depth research on new drugs.

An ethanol extract of NI rhizomes was found to possess strong nematicidal activity against two species of nematodes, Bursaphelenchus xylophilus and Meloidogyne incognita [89]. Four constituents isolated from the ethanol extract were identified as columbianetin, falcarindiol, falcarinol, and isoimperatorin. Among the four isolated constituents, two acetylenic compounds, falcarindiol and falcarinol (2.20–12.60 μg/mL and 1.06–4.96 μg/mL, respectively) exhibited stronger nematicidal activity than the two furanocoumarins, columbianetin, and isoimperatorin (21.83–103.44 μg/mL and 17.21–30.91 μg/mL, respectively) against the two species of nematodes, *B. xylophilus* and *M. incognita*.

Ding et al. found that an extract of NI could mitigate ovariectomy-induced osteoporosis in rats [90], which showed dose-dependent inhibited bone mineral density reduction of L4 vertebrae and femurs (*p* < 0.05). These findings suggest a promising avenue for developing alternative osteoporosis therapies from traditional medicinal plants, potentially benefiting patients with limited treatment options.

## 5. Conclusions

In this review, we have described the Chinese endemic herb genus *Notopterygium*’s complex phytochemical components as well as its various pharmacological profiles, particularly focusing on NI and NF. The discovery of more than 500 chemicals, particularly in the roots, has provided insight into their ability to potentially treat a range of illnesses, including inflammation and cancer. Although traditional applications have long recognized the advantages of these chemicals, the understanding of the mechanisms by which they produce their effects is still being revealed [81,98]. Insufficient research has been conducted on the phytochemistry and pharmacology of other species belonging to the *Notopterygium* genus. Additional endeavors are required to address this knowledge gap. The intricate relationship between *Notopterygium*’s broad effects and its chemical constituents, especially in connection with how different components bring about various therapeutic effects, warrants further investigation [99]. In order to fully utilize the therapeutic benefits of *Notopterygium*, it is crucial to advance the creation of more sophisticated screening models [100,101]. In the future, constructing activity screening models guided by the pharmacological effects of *Notopterygium*, discovering new active ingredients and studying their pharmacological mechanisms will become the main development directions of *Notopterygium* medicinal research.

## Figures and Tables

**Table 1:** The summary of biological activities and the related component/compounds of *Notopterygium* species.

Biological Activity	Component/Compound	Results	References
Anti-inflammatory	Notopterol	Down-regulated the hypersecretion of inflammatory mediators and alleviated the degradation of the extracellular matrix.	[68]
		Inhibited synthesis of inflammatory mediators such as IL-1β, IL-32, and IL-8 in LPS-stimulated human gingival fibroblasts.	[69]
		Enhanced the survival rate and improved the functioning of the right ventricle.	[70]
		Acted as therapeutic agent in the treatment of osteoarthritis (OA.)	[71]
	Pterostilbene and notopterol	Exhibited potential therapeutic effects on obstructive sleep apnea syndrome (OSAS).	[72]
	4-methyl-3-trans-hexenylferulate, (-)-boroylferulate, 4-methoxyphenyl ferulate, and phenylyl ferulate	Inhibited NO production in RAW 264.7 cells.	[73]
	3-hydroxy allyl polyacetylenes		[74]
	Notoflavinols A and B, and (2R)-5,4′-dihydroxy-7-O-[(E)-3,7-dimethyl-2,6-octadienyl]flavanone		[35,57]
	Nodakenetin	Reduced CFA-induced inflammatory pain, but did not have a substantial therapeutic impact.	[75]
	Falcarindiol	Activated the Nrf2/ARE pathway and induced cytoprotective enzymes.	[76]
	Polyacetylenes	Acted as selective partial PPARγ agonist in the luciferase reporter model.	[51]
	Volatile oils	Exhibited strong antioxidant activity in vitro and potent anti-inflammatory activity in zebrafish embryos.	[13]
	Extract of NI or the formula containing NI	Down-regulated P2X1, P2X3, P2X4, P2X5, and P2X7 to inhibit FCA-induced RA in rats.	[77]
		Regulated NLRP3, pro-Caspase-1, Caspase-1, and CD11b in the ankle joint of AA rats.	[78]
		Improved cognitive dysfunction in Alzheimer’s disease (AD) mice.	[79]
		Reduced levels of TNF-α, IL-6, and VEGF—possessed evident anti-arthritic effects in AIA rats.	[80]
		Regulated gut microbiota, inhibited pro-inflammatory factors in rats with IS.	[81]
Anti-tumor	Notopol, notopterol, 5-[(2 E,5 Z)-7-hydroxy-3,7-dimethyl-2,5-octadienoxy]psoralene, and 5-[(2,5)-epoxy-3-hydroxy-3,7-dimethyl-6-octenoxy]psoralene	Exhibited antiproliferative activity against hepg-2 and C6 cancer cell lines.	[82]
	Notopterol	Suppressed the viability, migration, and invasion capacity of the human HCC hepj5 and Mahlavu cell lines.	[83]
		Bound Janus kinase (JAK)2 and JAK3 kinase domains to inhibit JAK/signal transducers and activators of transcription (JAK-STAT) activation.	[84]
		Suppressed IL-17-induced proliferation and invasion of A549 lung adenocarcinoma cells via modulation of STAT3, NF-κB, and AP-1 activation.	[85]
		Exhibited anti-glioma effects and alleviated neuropsychiatric symptoms.	[86]
	Strustin	Exhibited activity on PANC-1 (PC_50_ = 7.2 μmol/L) and PSN-1 (PC_50_ = 7.8 μmol/L).	[87]
	Ostruthin and (-)-bornyl ferulate	Induced comparable neurite-like structures in twenty percent of rat PC12 cells at 2 mg/mL, and showed cytotoxicity at concentrations higher than 3 mg/mL.	[87]
Antibacterial	Volatile oils	The diameters of inhibition zone (dizs) of NI-EO against *E. coli* and *S. aureus* were 14.63 and 11.25 mm and the minimum inhibitory concentrations were 3.75 and 7.5 μL/mL, respectively.	[13]
Antifungal	Ethyl acetate extract of NI	Exhibited antifungal activities against apple fruit pathogens of Colletotrichum *gloeosporioides* and Botryosphaeria dothidea with MIC values ranging from 8 to 250 mg L^−1^.	[46]
The others	Extract of NI	Showed potential therapeutic effects on ovariectomy-induced osteoporotic and thrombus rats.	[88]
	Columbianetin, falcarindiol, falcarinol, and isoimperatorin	Showed strong nematicidal activity against the two species of nematodes.	[89]
	Extract of NI	Mitigated ovariectomy-induced osteoporosis in rats.	[90]
